# Investigating the Influence of Inverse Preferential Attachment on Network Development

**DOI:** 10.3390/e22091029

**Published:** 2020-09-15

**Authors:** Cynthia S. Q. Siew, Michael S. Vitevitch

**Affiliations:** 1 Department of Psychology, National University of Singapore, Singapore 117570, Singapore; 2 Department of Psychology, University of Kansas, Lawrence, KS 66045, USA; mvitevit@ku.edu

**Keywords:** network growth, preferential attachment, inverse preferential attachment, language networks, language development

## Abstract

Recent work investigating the development of the phonological lexicon, where edges between words represent phonological similarity, have suggested that phonological network growth may be partly driven by a process that favors the acquisition of new words that are phonologically similar to several existing words in the lexicon. To explore this growth mechanism, we conducted a simulation study to examine the properties of networks grown by inverse preferential attachment, where new nodes added to the network tend to connect to existing nodes with fewer edges. Specifically, we analyzed the network structure and degree distributions of artificial networks generated via either preferential attachment, an inverse variant of preferential attachment, or combinations of both network growth mechanisms. The simulations showed that network growth initially driven by preferential attachment followed by inverse preferential attachment led to densely-connected network structures (i.e., smaller diameters and average shortest path lengths), as well as degree distributions that could be characterized by non-power law distributions, analogous to the features of real-world phonological networks. These results provide converging evidence that inverse preferential attachment may play a role in the development of the phonological lexicon and reflect processing costs associated with a mature lexicon structure.

## 1. Introduction

Many complex systems, such as the Internet, brain networks, and social networks, can be classified as networks—collections of entities connected to each other in a web-like fashion—permitting the application of network analysis to study these systems (see [1] for a review). A common feature across diverse complex networks is their scale-free degree distribution, whereby most nodes in the network have very few edges or links and a few nodes have many edges or links. Preferential attachment models of network growth, where new nodes that are added to the network tend to connect to existing nodes with many links (i.e., high degree nodes), have been prominent in the literature covering network growth and evolution, because such models describe a generic mechanism that provides an elegant account of the emergence of scale-free complex networks [2,3,4,5]. In this paper, we conducted a series of network simulations to specifically examine the properties of networks grown via a different mechanism, which we refer to as inverse preferential attachment, where new nodes added to the network tend to connect to existing nodes with fewer edges.

Our present approach of simulating network growth via inverse preferential attachment was directly motivated by recent research examining the development of language networks constructed from phonological similarity among words. In these language networks, nodes represent words, while edges are placed between words that share similar sounds [6]. Previous research has shown that the structure of the phonological lexicon has measurable influences on various language-related processes [7,8,9]. Research investigating the processes that facilitate the acquisition of the phonological form of a word indicate that phonological network growth may be driven by alternative network growth mechanisms other than the widely studied preferential attachment [10,11,12]. Central to the present study is a recent paper by Siew and Vitevitch [12], who conducted a longitudinal analysis of phonological networks of English and Dutch words and found that preferential attachment was a better predictor of acquisition than preferential acquisition. Furthermore, although the standard preferential attachment model was a significant predictor of acquisition at early stages of network growth (i.e., when the phonological network was “young”), there was a subsequent shift in the network growth mechanism, such that an inverse variant of preferential attachment became a significant predictor of acquisition at later stages of network growth (i.e., when the phonological network matured and contained many nodes and edges). To put it in another way, a network growth mechanism that prioritized the learning of words that were phonologically similar to words with many phonological neighbors (i.e., many edges) in the lexicon was important in the early stages of development, whereas a growth mechanism that prioritized the learning of words that were phonologically similar to words with few phonological neighbors (i.e., few edges) in the lexicon was important in the later stages of development. Siew and Vitevitch [12] provided further empirical support for inverse preferential attachment by conducting a word learning experiment, which found that people with mature lexicons (i.e., college students) were able to better learn made-up words that were phonologically similar to words with few phonological neighbors in the lexicon, as compared to made-up words that were phonologically similar to words with many phonological neighbors.

Given these intriguing patterns of phonological network growth observed in our prior work, the aim of the present paper was to conduct a computational exploration of these patterns. To this end, we conducted a series of network growth simulations to examine if networks generated by the preferential attachment growth algorithm and its inverse variant, as well as combinations of each algorithm, might lead to structurally different networks. Even though we examine a simple model of network growth here, this has potentially important theoretical implications for understanding how the large-scale development of the phonological lexicon could occur. For instance, the artificial randomly grown networks examined by Callaway and colleagues [13] exhibited many network characteristics that were also observed in real phonological networks [6], and we wanted to investigate if simulating network growth with typical or inverse preferential attachment mechanisms may also lead to networks with characteristics observed in real phonological networks. Computing network measures (such as average shortest path length, network diameter) and degree distributions is one way of evaluating the structure of simulated networks. Network measures such as the average shortest path length and network diameter provide an indication of the overall efficiency of the network (i.e., efficiency referring to a network’s ability to quickly exchange information or for activation to spread in a network [14]), whereas degree distributions can be considered as structural signatures of the network, which can inform us about the growth processes that gave rise to the network [2]. If the overall network measures of simulated networks are qualitatively similar to real-world phonological language networks, this suggests that growth mechanisms that gave rise to the observed structure of the simulated networks might also contribute to the acquisition of phonological representations.

It is important to acknowledge that the approach taken here does not provide conclusive proof that either one of these network growth algorithms is entirely responsible for producing the structures observed in real-world phonological networks. Indeed, much research (e.g., [15]) has demonstrated that the famed scale-free network, for example, can be produced not only by the preferential attachment algorithm proposed by Barabási and Albert [2], but also by a number of other methods as well. In the absence of any other information, it would indeed be unwise to assert that a particular algorithm was responsible for producing a network with a particular set of characteristics. In the domain of psycholinguistics, however, there is a long and rich history of research that provides some guidance on which possible algorithms are unattested in the languages of the world, and therefore not plausible as a mechanism for the acquisition of words; or which algorithms have been observed with other research methods (e.g., case studies, archival analyses, laboratory-based experiments), and therefore might be plausible mechanisms for the acquisition of words and may also provide insight into certain language disorders (e.g., [16]). We performed the present simulation merely to offer an additional piece of evidence to complement the archival analyses and experiments in our earlier work [12], which might help to constrain the realm of possibility to the more restricted space of plausibility.

## 2. Materials and Methods

Each simulation began with a single node. The growth of the network was simulated by adding a new node and a single new link to the network at each iteration. Each simulation continued for 999 iterations, such that each resulting network consisted of 1000 nodes and 999 edges. To simulate the growth of the network via preferential attachment, the probability that a new node connected to a given existing node was proportional to the number of connections that the existing node had to other nodes in the network. Therefore, a new node was more likely to connect to an existing node with a high degree. To simulate the growth of the network via inverse preferential attachment, the probability that a new node connected to a given existing node was inversely proportional to the number of connections that the existing node had to other nodes in the network. In this case, a new node was more likely to connect to an existing node with a low degree. Finally, in random attachment, the new node had an equal probability of connecting to any existing node, regardless of its degree.

**Figure 1:**
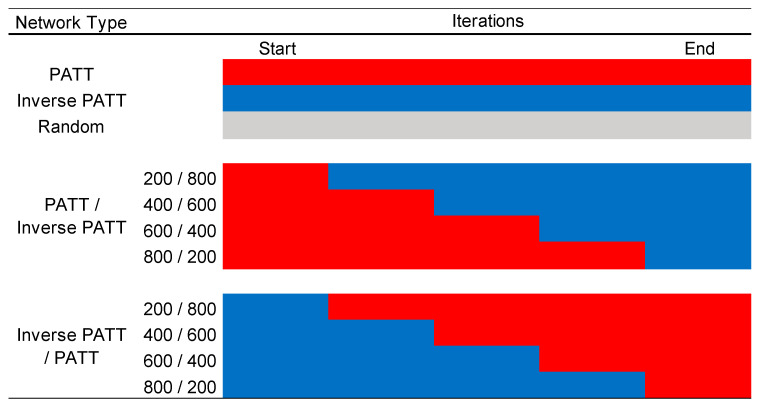
A summary of the 11 network growth conditions simulated in the present study. Red cells indicate growth by standard preferential attachment, blue cells indicate growth by inverse preferential attachment. PATT, preferential attachment.

There was a total of 11 different network types, i.e., networks that were grown by different mechanisms and by various combinations of those mechanisms (see Figure 1 for a summary). Three network types were generated by a single mechanism, i.e., entirely via preferential attachment (PATT), entirely via inverse preferential attachment (iPATT), and via random attachment (Random). For ease of exposition, PATT refers to networks generated by preferential attachment and iPATT refers to networks generated by inverse preferential attachment. The remaining 8 network types were generated using a combination of preferential attachment and inverse preferential attachment, to explore how the “blending” of different growth models affected the development of the network, given that Siew and Vitevitch [12] found that preferential attachment was influential earlier in development but not later in development. Of these 8 network types, four were generated via preferential attachment first (for 200, 400, 600, and 800 iterations) followed by the inverse variant for the remainder of the iterations, and four were generated via inverse preferential attachment first (for 200, 400, 600, and 800 iterations) followed by the original preferential attachment model for the remainder of the iterations. The network growth simulations were repeated 100 times for each network type, resulting in a total of 1100 simulated networks. All simulations were conducted in R using the igraph library [17]. Analyses of the final network structure and their degree distributions were also conducted in R using the igraph and poweRlaw [18] libraries, respectively. The simulation and analysis R scripts, as well as the simulated network data, are available via the Supplementary Materials. 

The characteristics of the 1100 simulated networks can be quantified in various ways to examine how the overall structures of these networks differ across different simulation conditions (i.e., network type). The following network measures will be computed: average shortest path length, network diameter, and degree distribution.

The shortest path length between two nodes refers to the fewest number of links that must be traversed to get from one node to another node in the network. The average shortest path length (ASPL) is the mean of the shortest path length obtained from every possible pairing of nodes in the network. A closely related measure is the diameter of the network; this is the longest shortest path length that exists in the network. The degree distribution refers to the probability distribution of node degrees in the network; in other words, how many nodes have a given number of connections in the network. Recall that degree refers to the number of connections incident to a node.

## 3. Results

### 3.1. Overall Network Characteristics of Simulated Networks

For each of the simulated networks, the average shortest path length and network diameter was computed. Table 1 shows the mean ASPL and network diameter for the networks in each condition (i.e., network type) of the simulations.

**Figure 2:**
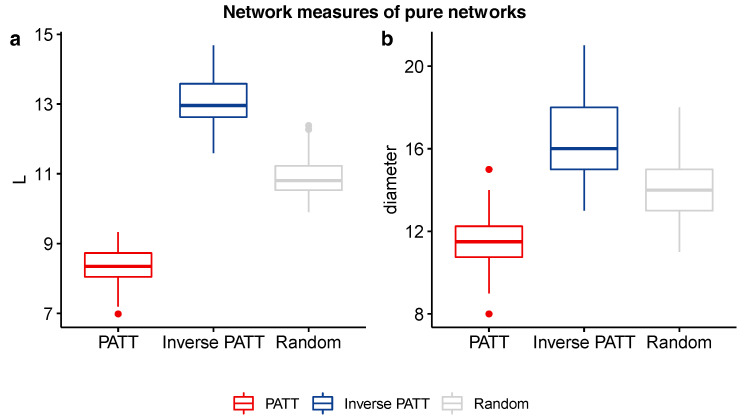
Boxplots of ASPL (**a**) and network diameter (**b**) values of networks grown via preferential attachment (PATT), inverse preferential attachment (Inverse PATT), and random attachment (Random).

**Figure 3:**
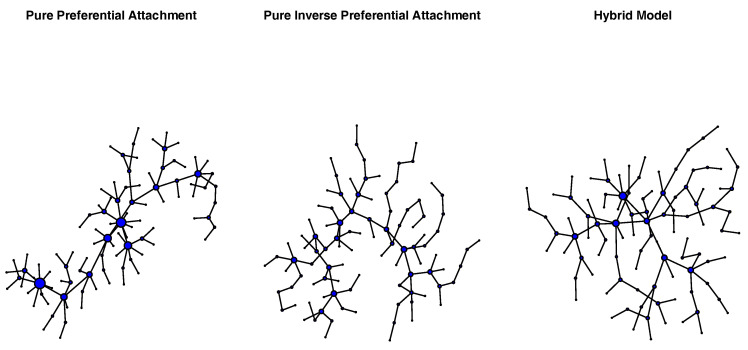
Network visualizations of exemplar networks generated via pure preferential attachment (**left**), pure inverse preferential attachment (**center**), and preferential attachment followed by inverse preferential attachment (hybrid model; **right**). Each network consisted of 100 nodes. The size of each node reflects its degree.

Independent samples *t*-tests comparing the average shortest path length and network diameter of PATT and iPATT networks showed that iPATT networks had larger diameters (*t*(189.76) = 59.47, *p* < 0.001) and longer ASPLs (*t*(187.7) = 21.27, *p* < 0.001) than PATT networks (see Figure 2). This suggests that networks generated by preferential attachment tend to be denser and more compact as compared to networks generated by inverse preferential attachment, despite having the same numbers of nodes and edges (see Figure 3 for network visualizations of the overall network structures).

**Figure 4:**
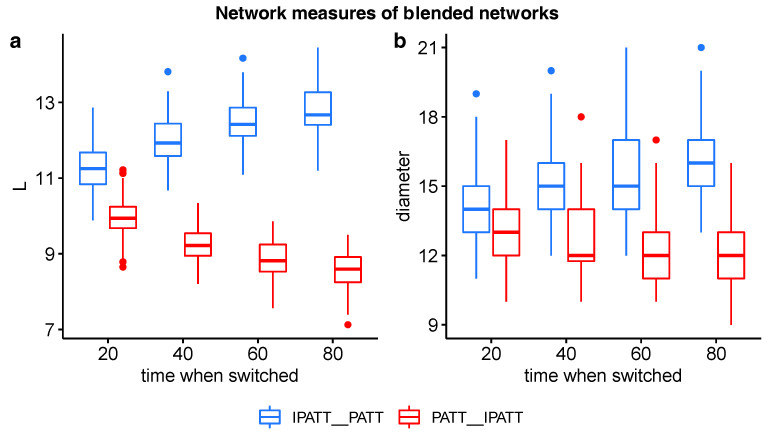
Boxplots of ASPL (**a**) and network diameter (**b**) values of networks grown via blends of preferential attachment and inverse preferential attachment. The x-axis indicates the percentage proportion of nodes added before the network algorithm was switched. Red bars indicate networks first grown by PATT followed by inverse PATT. Blue bars indicate networks first grown by inverse PATT followed by PATT.

Interestingly, the networks grown by PATT first followed by iPATT tended to have smaller diameters and shorter average shortest path lengths than the networks grown by iPATT first followed by PATT, regardless of when the growth model “switched” to the other growth model (see Figure 4). This observation is supported by significant the interaction effects of the network type (PATT–inverse PATT; inverse PATT–PATT) and time of switch (20%, 40%, 60%, 80% of nodes added) for ASPL (*F*(1, 796) = 761.04, *p* < 0.001) and diameter (*F*(1, 796) = 91.68, *p* < 0.001) in a between-group two-way analysis of variance. This result may suggest that networks generated by the preferential attachment growth algorithm at the initial stages (even for a short period) may be more navigable than networks that are generated by the inverse preferential attachment growth algorithm at the initial stages.

### 3.2. Degree Distributions

In this section, we examine the degree distributions of networks generated in Section 2. Raw counts of the numbers of nodes with degrees of various values were obtained from each network. In part 1, a power law was first fitted to the degree distributions and the goodness-of-fit of the power law to the data was evaluated via a bootstrapping approach. In part 2, the data were fit to alternative distributions (log-normal, exponential, and Poisson distributions) and tests were conducted to assess the fit of the power law to the data as compared to alternative distributions. This sequence of analyses closely follows the recommendations of Clauset, Shalizi, and Newman [19] for analyzing power law-distributed data in a statistically rigorous manner (see [18] for more information on how to implement this analysis pipeline).

#### 3.2.1. Test for Power Law Fits via Bootstrapping

A power law was fit to the degree distributions of each of the simulated networks. Specifically, a power law was fit to the data and the scaling parameter, α (i.e., the exponent of the power law), was computed for a given x_min_ value (the minimum value for which the power law holds; see the x_min_ and α columns in Table 2). Note that all exponents were <2, lower than what is usually observed in real-world networks, where 2 < α < 3 [19]. This may be due to the simplicity of the simulations conducted (i.e., only 1 node and 1 edge were added to the network at each iteration), which led to sparser networks.

As suggested by Clauset et al. [19], we evaluated whether the observed degree distributions actually followed a power law via a bootstrapping approach. Specifically, 1000 degree distributions were sampled from the empirical degree distribution of interest, a power law was fit to that degree distribution, and the exponent was computed. Mean α indicates the mean exponent of the 1000 bootstrapped networks and SD α indicates the standard deviation of the 1000 bootstrapped networks. A goodness-of-fit test was then conducted to determine if the exponent obtained from the original degree distribution was likely to have come from the bootstrapped “population” of exponents. As the point estimate *p*-values were not significant (all *p*-values > 0.05), this indicated that for all 11 network types, the power law distribution provided a plausible fit to the degree distributions (i.e., the exponent estimate is stable despite random fluctuations). Table 2 shows a summary of the results of the goodness-of-fit test for all 11 network types (see the mean α, SD α, Kolmogorov–Smirnov statistics, and *p*-value columns).

Although the results of the bootstrap seem to suggest that both degree distributions from the PATT and iPATT networks followed a power law, a closer look at Table 2 indicates that the Kolmogorov–Smirnov statistic for the iPATT network (*D* = 0.25) was larger than the Kolmogorov–Smirnov statistic for the PATT network (*D* = 0.11). The magnitude of *D* is an indicator of the “distance” between the fitted distribution and the actual data. In this case, the degree distribution of the network that was simulated via inverse preferential attachment deviated to a greater extent from a power law as compared to the network that was simulated via preferential attachment. This was confirmed by a visual inspection of the cumulative degree distributions (see Figure A3 in the Appendix A).

#### 3.2.2. Statistical Comparison with Other Degree Distributions

As recommended by Clauset et al. [19], another way of investigating the nature of degree distributions in networks is to fit alternative distributions (exponential, log-normal, and Poisson distributions) to the degree distributions of all networks and conduct the relevant goodness-of-fit tests to compare the fit of these distributions to the fit of the power law to the data. The comparison of the power law and these distributions constitutes a non-nested model comparison, so Vuong’s test of non-nested hypotheses was used instead of the likelihood ratio test (for details, please see [20]). Vuong’s test computes a *V*-statistic, one-sided *p*-value, and two-sided *p*-value. The one-sided *p*-value indicates the probability of obtaining the particular value of log likelihood ratio if the power law is not true. In other words, a significant one-sided *p*-value indicates that the power law distribution is a good fit to the data (low probability that the alternate distribution could account for the data), whereas a non-significant one-sided *p*-value indicates that the power law distribution is a not good fit to the data (high probability that the alternate distribution could account for the data). The two-sided *p*-value indicates the probability that both distributions being compared are equally “distant” from the data. In other words, a significant two-sided *p*-value indicates that one distribution is a significantly better fit to the data than the other distribution, whereas a non-significant two-sided *p*-value indicates that neither distribution is preferred.

**Figure A1:**
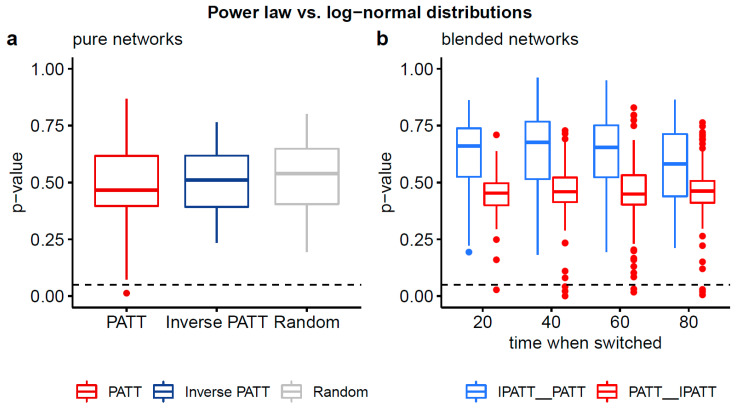
Boxplots of two-sided *p*-values from Vuong’s test of non-nested models comparing the fits of power law and log-normal distributions to the degree distributions of simulated networks. Panel (**a**) compares the degree distributions of pure networks and panel (**b**) compares the degree distributions of blended networks. Non-significant *p*-values (based on an alpha-level of 0.05) indicate that neither distribution is preferred.

**Figure A2:**
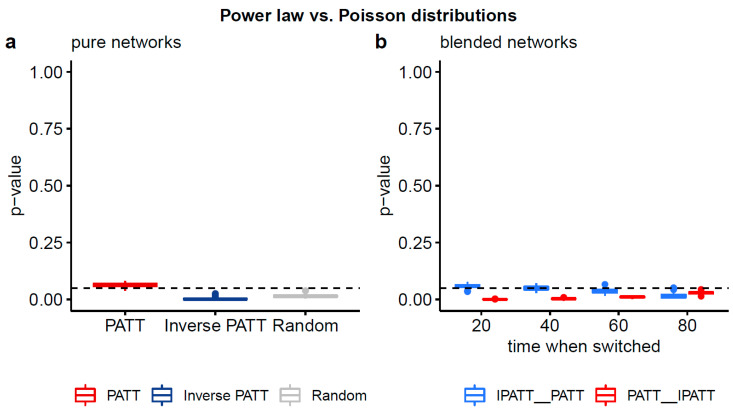
Boxplots of two-sided *p*-values from Vuong’s test of non-nested models comparing the fits of power law and Poisson distributions to the degree distributions of simulated networks. Panel (**a**) compares the degree distributions of pure networks and panel (**b**) compares the degree distributions of blended networks. Significant *p*-values (based on an alpha-level of 0.05) indicate that one distribution fits the empirical data better. Based on the one-sided *p*-values (see Table A1), the power law distribution provides a better fit than the Poisson distribution.

**Figure A3:**
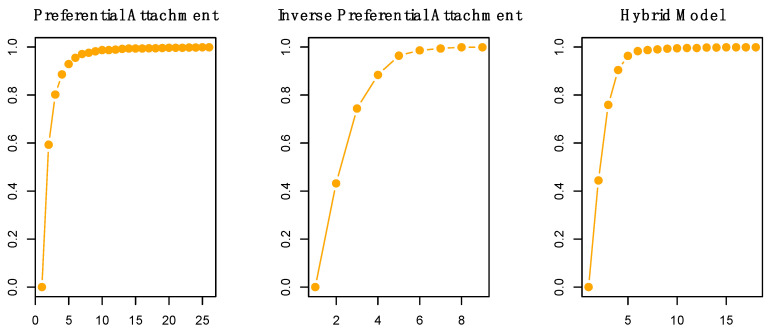
Cumulative degree distributions of exemplar networks generated via pure preferential attachment (**left**), pure inverse preferential attachment (**center**), and preferential attachment followed by inverse preferential attachment (hybrid model; **right**).

The results of these comparisons are summarized in Table 3 below, with more detailed statistics available in Table A1 of Appendix A. For the power law and Poisson comparison, the significant two-sided *p*-values and significant one-sided *p*-values for all 11 network types indicate that a power law distribution was a significantly better fit to the data than a Poisson distribution. For the power law and log-normal comparison, the non-significant two-sided *p*-values for all 11 network types indicate that one distribution cannot be favored over the other. See Figure A1 and Figure A2 in the Appendix A for a visual depiction of these results.

**Figure 5:**
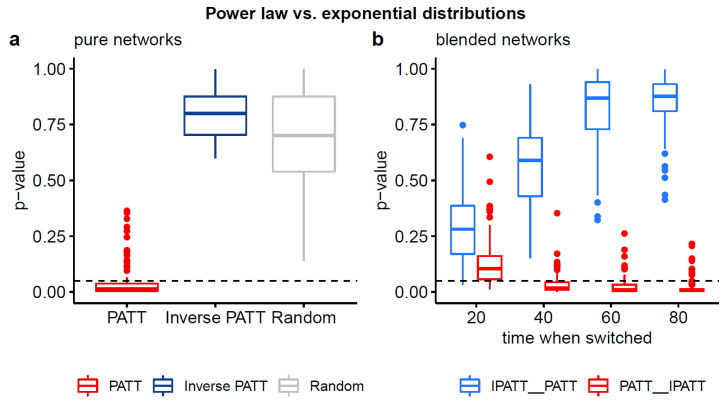
Boxplots of two-sided *p*-values from Vuong’s test of non-nested models comparing the fits of power law and exponential distributions to the degree distributions of simulated networks. Panel (**a**) compares the degree distributions of pure networks and panel (**b**) compares the degree distributions of blended networks. Non-significant *p*-values (based on an alpha-level of 0.05) indicate that neither distribution is preferred. Significant *p*-values (based on an alpha-level of 0.05) indicate that one distribution fits the empirical data better. Based on the 1-sided *p*-values (see Table A1), the power law distribution provides a better fit than the exponential distribution for all networks grown by preferential attachment, except for when the switch to its inverse variant occurs early.

The comparison between the power law and exponential distribution is more informative (see Figure 5). For the PATT network, the two-sided *p*-value was significant, indicating that the two distributions were not equivalent in terms of their fit to the data, with one distribution being a better fit. The results of the one-sided test indicate that the power law was a better fit for the degree distribution generated by the preferential attachment as compared to an exponential distribution. For the iPATT and random network, the two-sided *p*-value was not significant, indicating that the two distributions (power law and exponential) were equivalent in terms of their fit to the data.

Turning to the results of Vuong’s test for the combination (i.e., blended) networks, we observe that for all network types generated with iPATT followed by PATT, the two-sided *p*-values for the power law and exponential comparison were non-significant, indicating that the two distributions were equivalent in terms of their fit to the data, similar to the iPATT-only and random networks. In contrast, the pattern of results varied for the networks generated with PATT followed by iPATT. The network where the first 200 iterations were based on the PATT model had two-sided *p*-values that were non-significant (similar to the iPATT-only network), whereas the other networks where the first 400, 600, and 800 iterations were based on the PATT model had two-sided and one-sided *p*-values that were significant, indicating that the power law was a better fit than the exponential distribution (similar to the PATT only network).

In summary, the key finding of the analyses of the network structure and degree distributions was that the blended network that was first generated by PATT followed by iPATT led to (i) a network structure with relatively low values for the ASPL and diameter (i.e., low values of ASPL and diameter in Figure 4a,b) and (ii) degree distributions that could not be exclusively classified as a power law (i.e., *p*-values > 0.05 in Figure 5b)—qualitatively resembling the properties of real-world phonological networks [21].

## 4. Discussion

The key finding from the simulations was that a model where the network was first generated by PATT for a short period (the first 200 out of 1000 iterations) before switching to the iPATT growth mechanism led to a network structure that was (i) more densely connected than if the growth models were reversed (i.e., smaller diameters and ASPL) and (ii) had a degree distribution that could be accounted for by alternative distributions (i.e., the exponential distribution that provided similar fits to the data as did the power law).

Recall that Siew and Vitevitch [12] found through an archival analysis and laboratory-based experiments that novel words that connected to existing words with few phonological neighbors in the lexicon were more likely to be learned than novel words that connected to existing words with many connections at later stages of development. We suggested that this switch may arise due to the increased processing costs associated with navigating a lexicon with a crowded phonological space [22], as well as the increased pressures on lexical representations to be better differentiated from each other in a more densely connected phonological lexicon (see the lexical restructuring hypothesis; [23,24]). We wished to explore these intriguing ideas computationally and simulated networks that were generated by a blend of different network growth mechanisms. Our results suggest that it is possible that the development of the phonological network may be better captured, at least partly, by an alternative network growth algorithm.

Overall, the simulations suggest that a particular combination of the PATT and iPATT network growth algorithms (i.e., the network that is initially “grown” by PATT followed by inverse PATT) led to the emergence of network characteristics that are suggestive of increased efficiencies in network navigation [25] (i.e., lower ASPL and smaller diameter) and degree distributions that are not necessarily best captured by a pure power law (i.e., not a purely scale-free degree distribution). We observed this in the case where the network was generated with PATT driving the initial stage for a short period (200 out of 1000 iterations) and iPATT driving the later stages of growth. This led to a network structure that was more densely connected than if the order of the growth models was reversed (i.e., iPATT followed by PATT) and a degree distribution that could be accounted for by alternative non-power law distributions, such as the exponential distribution, rather than if preferential attachment persisted for a longer period of time at the beginning (i.e., PATT continued for a longer period before the switch to iPATT occurred in the simulations).

Although small, simple networks were simulated in this study, the present findings nevertheless provide a proof-of-concept that the new growth principle that we proposed—inverse preferential attachment—can produce a degree distribution that is not necessarily captured by a power law and still lead to the emergence of network characteristics that facilitate efficient navigation (i.e., small diameter and low ASPL). These network features are qualitatively similar to the network features observed in real-world phonological networks [6,21]. In addition, we wish to highlight that the present analyses do not provide evidence that only the preferential attachment or inverse preferential attachment mechanisms are directly influencing the network structure of the phonological lexicon. What these results do suggest is that a countably infinite list of complicated and detailed constraints that capture the microscopic details of language may not be necessary to produce the structure observed in the phonological network. Rather, a simple assumption, such as the assumption examined mathematically by Callaway et al. [13]—stating that newly added nodes do not necessarily need to be attached to an existing node in the network—may lead to some of the structural features of the phonological network, such as the presence of lexical hermits in the phonological lexicon as observed by [6]. The results of the present simulation in conjunction with the long and rich history of research in psycholinguistics also allows us to constrain our search of possible algorithms involved in the acquisition of words to the space of plausible algorithms. Furthermore, the results of the present simulation lend credence to the idea that the principles that affect word learning may change over time as the lexicon becomes more “crowded” with similar sounding words or other cognitive constraints begin to exert an influence on acquisition (for similar influences on semantics, see [26]).

Finally, our results provide new avenues for research within the field of network science. First, although network scientists have previously examined the influence of constraints of costs on network growth (e.g., financial or space limitations on the expansion of air transportation networks [27]), the present findings suggest that it may also be important to consider how different costs introduced at different time-points of development shape future network growth. Second, network scientists commonly view network growth as operating via a process that maximizes node fitness [3,5]. In the case of preferential attachment and close variants of this model, the fitness of an individual node (i.e., its ability to gain new edges) is maximized by attaching to a high-degree node. The present findings suggest that understanding network growth requires a careful consideration of the functional purpose of each complex network. In the case of phonological network development, prioritizing the acquisition of new words that occupy sparser, peripheral areas of the phonological space at later stages of development when the core of the lexicon is already highly filled out may be especially important to increase the overall fitness and efficiency of the entire network. This provides accurate coverage of the entire phonological space in order to attain an overall network structure that is optimized for language processing. In other words, network growth may not be only about maximizing the fitness of individual nodes, but may also leverage on different types of network growth algorithms (such as inverse preferential attachment) to maximize the fitness of the network as a whole in order to facilitate the processes and operations that occur within that network.

## Figures and Tables

**Table 1:** Means and standard deviations of network measures of simulated networks, summarized by each of the 11 simulation conditions. Note that all simulated networks had the same number of nodes and edges (1000 nodes and 999 edges).

Network		Nodes	Edges	ASPL	Diameter
PATT	M	1000	999	8.34	11.55
	SD	0	0	0.50	1.42
Inverse PATT	M	1000	999	13.07	16.42
	SD	0	0	0.62	1.80
Random	M	1000	999	10.91	13.81
	SD	0	0	0.52	1.54
PATT–Inverse PATT					
**200/800**	**M**	**1000**	**999**	**9.97**	**13.27**
	**SD**	**0**	**0**	**0.50**	**1.47**
400/600	M	1000	999	9.22	12.58
	SD	0	0	0.49	1.58
600/400	M	1000	999	8.83	12.29
	SD	0	0	0.49	1.43
800/200	M	1000	999	8.55	11.93
	SD	0	0	0.49	1.50
Inverse PATT–PATT					
200/800	M	1000	999	11.25	14.28
	SD	0	0	0.59	1.60
400/600	M	1000	999	12.00	15.17
	SD	0	0	0.61	1.63
600/400	M	1000	999	12.48	15.59
	SD	0	0	0.59	1.76
800/200	M	1000	999	12.81	15.92
	SD	0	0	0.62	1.74

Legend: M = mean; SD = standard deviation; ASPL = average shortest path length; PATT = preferential attachment.

**Table 2:** Power law scaling parameter estimates and uncertainty estimates from the bootstrap procedure. Note that the bootstrap procedure was conducted for each simulated network; the means and standard deviations of estimates are shown in the table.

	x_min_		α		Mean Bootstrapped α		SD Bootstrapped α		*KS*-Statistic		*p*-Value	
Network	M	SD	M	SD	M	SD	M	SD	M	SD	M	SD
PATT	2.09	2.27	1.45	0.06	1.55	0.08	0.36	0.14	0.11	0.02	0.74	0.16
Inverse PATT	9.98	10.97	1.42	0.15	1.70	0.24	0.74	0.26	0.25	0.02	0.34	0.08
Random	10.51	13.51	1.49	0.17	1.68	0.20	0.67	0.23	0.19	0.02	0.52	0.15
PATT/Inverse PATT												
200/800	1.14	0.51	1.34	0.03	1.47	0.05	0.40	0.07	0.16	0.02	0.60	0.13
400/600	1.13	0.40	1.39	0.03	1.51	0.04	0.40	0.08	0.12	0.02	0.72	0.18
600/400	1.28	0.80	1.41	0.04	1.51	0.05	0.37	0.09	0.11	0.02	0.76	0.16
200/800	1.34	0.89	1.42	0.04	1.52	0.05	0.35	0.09	0.11	0.02	0.73	0.19
Inverse PATT/PATT												
200/800	8.50	6.87	1.56	0.13	1.72	0.15	0.63	0.18	0.15	0.02	0.70	0.20
400/600	18.07	14.73	1.64	0.20	1.84	0.21	0.81	0.26	0.17	0.02	0.67	0.19
600/400	23.40	20.21	1.65	0.25	1.87	0.26	0.86	0.28	0.20	0.02	0.59	0.18
200/800	24.34	27.48	1.61	0.29	1.84	0.28	0.84	0.29	0.23	0.02	0.48	0.14

Legend: M = mean; SD = standard deviation; KS = Kolmogorov–Smirnov; PATT = preferential attachment.

**Table 3:** Summary of Vuong’s tests of non-nested models comparing power law distributions to alternative distributions (exponential, log-normal, Poisson). The cell indicates the preferred distribution from the comparison; n.d. indicates that no distribution can be favored.

Network	PL vs. Exp	PL vs. LN	PL vs. Pos
PATT	PL	n.d.	PL
Inverse PATT	n.d.	n.d.	PL
Random	n.d.	n.d.	PL
PATT/Inverse PATT			
**200/800**	**n.d.**	**n.d.**	**PL**
400/600	PL	n.d.	PL
600/400	PL	n.d.	PL
200/800	PL	n.d.	PL
Inverse PATT/PATT			
200/800	n.d.	n.d.	PL
400/600	n.d.	n.d.	PL
600/400	n.d.	n.d.	PL
200/800	n.d.	n.d.	PL

Legend: PL = power law; LN = log-normal; Pos = Poisson; Exp = exponential; PATT = preferential attachment; n.d. = no difference.

## Supplementary Materials

All relevant materials and programming scripts are available on the Open Science Framework (https://osf.io/e8gwr).

## Appendix A

The appendix contains additional detail in relation to the analyses of the degree distributions of the simulated networks.

**Table A1:** Degree distributions of simulated networks were fitted to exponential, log-normal, and Poisson distributions and compared against the fitted power law distribution using Vuong’s test of mis-specified, non-nested hypotheses. Note that each set of model comparisons was conducted for each of the 100 simulated networks per condition or network type. The present table displays the mean and standard deviations of the test statistic and *p*-values.

**Power Law Vs. Exponential**						
	*V*-statistic		2-sided *p*		1-sided *p*	
**Network**	**M**	**SD**	**M**	**SD**	**M**	**SD**
PATT	2.387	0.614	0.046	0.080	0.023	0.040
Inverse PATT	−0.156	0.251	0.800	0.113	0.560	0.098
Random	0.420	0.359	0.682	0.222	0.347	0.120
PATT/Inverse PATT						
200/800	1.614	0.399	0.135	0.111	0.067	0.056
400/600	2.320	0.483	0.037	0.048	0.018	0.024
600/400	2.597	0.578	0.025	0.041	0.012	0.021
200/800	2.626	0.511	0.021	0.039	0.011	0.020
Inverse PATT/PATT						
200/800	1.116	0.357	0.293	0.151	0.147	0.076
400/600	0.593	0.288	0.570	0.176	0.285	0.088
600/400	0.221	0.241	0.817	0.161	0.416	0.088
200/800	0.009	0.253	0.849	0.120	0.498	0.097
**Power law vs. Log-normal**						
	*V*-statistic		2-sided *p*		1-sided *p*	
**Network**	**M**	**SD**	**M**	**SD**	**M**	**SD**
PATT	−0.735	0.350	0.488	0.171	0.756	0.085
Inverse PATT	−0.695	0.227	0.498	0.137	0.751	0.069
Random	−0.672	0.267	0.517	0.160	0.742	0.080
PATT/Inverse PATT						
200/800	−0.777	0.201	0.446	0.091	0.777	0.045
400/600	−0.809	0.391	0.448	0.132	0.776	0.066
600/400	−0.798	0.340	0.450	0.154	0.775	0.077
200/800	−0.774	0.354	0.465	0.138	0.768	0.069
Inverse PATT/PATT						
200/800	−0.519	0.272	0.618	0.172	0.691	0.086
400/600	−0.500	0.272	0.631	0.176	0.685	0.088
600/400	−0.514	0.260	0.620	0.167	0.690	0.084
200/800	−0.590	0.270	0.570	0.171	0.715	0.085
**Power law vs. Possion**						
	*V*-statistic		2-sided *p*		1-sided *p*	
**Network**	**M**	**SD**	**M**	**SD**	**M**	**SD**
PATT	1.860	0.066	0.063	0.009	0.032	0.005
Inverse PATT	3.198	0.411	0.003	0.005	0.002	0.003
Random	2.453	0.151	0.015	0.006	0.008	0.003
PATT/Inverse PATT						
200/800	3.390	0.161	0.001	0.001	0.000	0.000
400/600	2.926	0.138	0.004	0.002	0.002	0.001
600/400	2.554	0.118	0.011	0.003	0.006	0.002
200/800	2.184	0.075	0.029	0.005	0.015	0.003
Inverse PATT/PATT						
200/800	1.899	0.072	0.058	0.009	0.029	0.005
400/600	1.967	0.084	0.050	0.009	0.025	0.005
600/400	2.103	0.118	0.037	0.010	0.018	0.005
200/800	2.460	0.240	0.017	0.012	0.008	0.006
